# Wild common crossbills produce redder body feathers when their wings are clipped

**DOI:** 10.1186/s40850-022-00150-9

**Published:** 2022-08-23

**Authors:** Blanca Fernández-Eslava, Alejandro Cantarero, Daniel Alonso, Carlos Alonso-Alvarez

**Affiliations:** 1 Department of Environmental Biology, Universidad de Navarra, 31008 Pamplona, Spain; 2 Department of Physiology, Veterinary School, Complutense University of Madrid, Avenida Puerta de Hierro s/n, 28040 Madrid, Spain; 3 Department of Ornithology, Aranzadi Sciences Society, Zorroagagaina 11, 20014 Donostia-S. Sebastián, Spain; 4 Department of Evolutionary Ecology, National Museum of Natural Sciences (MNCN), Spanish National Research Council (CSIC), C/ José Gutiérrez Abascal 2, 28006 Madrid, Spain

**Keywords:** Animal coloration, Carotenoid-based ornaments, Flying effort, Flight workload, Shared-pathway hypothesis, Sexual signaling

## Abstract

**Background:**

The animal signaling theory posits that conspicuous colorations exhibited by many animals have evolved as reliable signals of individual quality. Red carotenoid-based ornaments may depend on enzymatic transformations (oxidation) of dietary yellow carotenoids, which could occur in the inner mitochondrial membrane (IMM). Thus, carotenoid ketolation and cell respiration could share the same biochemical pathways. Accordingly, the level of trait expression (redness) would directly reveal the efficiency of individuals’ metabolism and, hence, the bearer quality in an unfalsifiable way. Different avian studies have described that the flying effort may induce oxidative stress. A redox metabolism modified during the flight could thus influence the carotenoid conversion rate and, ultimately, animal coloration. Here, we aimed to infer the link between red carotenoid-based ornament expression and flight metabolism by increasing flying effort in wild male common crossbills *Loxia curvirostra* (Linnaeus). In this order, 295 adult males were captured with mist nets in an Iberian population during winter. Approximately half of the birds were experimentally handicapped through wing feather clipping to increase their flying effort, the other half being used as a control group. To stimulate the plumage regrown of a small surface during a short time-lapse, we also plucked the rump feathers from all the birds.

**Results:**

A fraction of the birds with fully grown rump feathers (34 individuals) could be recaptured during the subsequent weeks. We did not detect any significant bias in recovery rates and morphological variables in this reduced subsample. However, among recaptured birds, individuals with experimentally impaired flying capacity showed body mass loss, whereas controls showed a trend to increase their weight. Moreover, clipped males showed redder feathers in the newly regrown rump area compared to controls.

**Conclusions:**

The results suggest that wing-clipped individuals could have endured higher energy expenditure as they lost body mass. Despite the small sample size, the difference in plumage redness between the two experimental groups would support the hypothesis that the flying metabolism may influence the redox enzymatic reactions required for converting yellow dietary carotenoids to red ketocarotenoids.

**Supplementary Information:**

The online version contains supplementary material available at 10.1186/s40850-022-00150-9.

## Background

Conspicuous colorations in many animal species have attracted the attention of evolutionary biologists from Charles Darwin. The current animal signaling theory posits that these traits may evolve as individual quality signals that act in sexual or social signaling contexts [1]. If evolving as individual quality signals, the traits should transmit reliable information favoring the receiver’s fitness [1]. It has been hypothesized that the signal reliability is maintained by the costs of trait production, preventing potential cheaters from correctly expressing the trait (i.e. handicap signals) [2–4]. Additionally, or alternatively, signal reliability could be assured when a close link between the trait development process and the signaler’s intrinsic quality is established. These traits are known as index signals [1]. The most easily recognizable index signals are some beetles’ huge weapons (mandibles), big antlers of ungulates and frog or deer vocalization frequencies, all strongly correlated to individual body size (e.g. [5]). All of them should allow inferring individual quality because body size determines individual fitness in these species [1].

In the last years, another index signal type has been proposed. It is produced by pigments (red keto-carotenoids) whose synthesis could be produced at the core of the cell respiratory function, i.e. into the mitochondrion [6–8]. We should first mention that, in many avian species, the red colorations are generated by converting yellow carotenoids acquired with food into red keto-carotenoids [7, 9]. That transformation involves redox enzymatic reactions [7, 9]. The cited change is performed by a group of enzymes (ketolases) that seems to be located at the inner mitochondrial membrane (IMM [7, 10];). This means they would share the biochemical pathways involved in cell respiration (i.e. the shared-pathway hypothesis; see [6, 7]). Thus, the level of trait expression (redness) would directly reveal the basis of metabolic function and, therefore, the bearer quality in an unfalsifiable way [6].

Otto Völker (1957 [11];) was probably the first to suggest that red carotenoid-based colorations are constrained by redox metabolism (see also [12, 13]). He studied male common crossbills (*Loxia curvirostra,* Linnaeus), whose plumage color ranges from dull yellow to bright red, and feather redness is due to feather ketocarotenoid accumulation [14, 15]. The researcher observed that the red males housed in cages could not replace their original red plumages and were always molted to produce yellow feathers. He suspected that, contrarily, crossbills allowed to fly could produce red feathers because they would have a better redox metabolism favoring the conversion of dietary yellow pigments (see also [13]).

Different avian studies have described that the flying effort may increase oxidative stress [16–18]. Oxidative stress is usually defined as an imbalance between the production rate of reactive oxygen species (ROS) due to cell metabolism and the state of different antioxidant defenses (e.g. [19]). It leads to oxidative damage, which is involved in senescence and age-related diseases [20]. An important part of ROS in cells is generated into the mitochondrion during cell respiration [21, 22]. The flying effort, manipulated by training or inferred from migratory behavior, was positively correlated to mitochondrial oxidative stress in birds’ blood and pectoral muscles [23, 24]. It has also been correlated to higher superoxide dismutase 2 (a specific mitochondrial antioxidant enzyme) gene expression in avian pectoral muscles [25]. Therefore, we can hypothesize that the redox metabolism modified during the flight could influence the carotenoid conversion rate and, ultimately, body coloration, such as early proposed by Völker [11].

However, the evidence to the present date is weak and mostly based on a few avian studies where flying effort was increased and color expression measured. First, Schmidt-Wellenburg et al. augmented the flying workload of captive Rosy starlings (*Pastor roseus*; Linnaeus) by using a wind tunnel [26]. Their pink plumage is generated by red ketocarotenoids [27]. They did not find any significant effect on plumage color after the natural molt [26]. Leclaire et al. also increased the flying effort of wild adult kittiwakes (*Rissa trydactila;* Stephens) but in this case by clipping some wing feathers during reproduction [28]. This technique has often been used to manipulate the cost of reproduction with different objectives (e.g. [29–31]). Leclaire et al. [28] reported a brightness decline in the red bill gape of kittiwakes (a trait also colored by ketocarotenoids: [32]). However, they did not detect changes in the hue or saturation of the gape, which are color parameters linked to tissue carotenoid concentrations [9]. Moreover, the red ketocarotenoids are obtained with food in the seabirds and directly deposited on ornaments without metabolic conversion (reviewed in [9]). Tarvin et al. performed a similar feather clipping in wild female American goldfinches (*Spinus tristis* Linnaeus) and did not detect bill color change when recaptured three weeks later [33]. However, red ketocarotenoids have still not been described in the bill of this species. Lastly, a fourth study indeed detected a change in the color of a ketocarotenoid-based ornament. Wild male red-backed fairy-wrens (*Malurus melanocephalus* Latham) with clipped wings increased the surface of red plumage after natural molt compared to controls [34], which would agree with Völker’s hypothesis (i.e. [11]). However, the wing-clipped birds reported better body condition (inferred from a body fat score) than controls. The authors thus deduced that the red surface increment was due to lower mobility or higher food intake. The birds could not have endured a higher flying effort. Note that body mass loss is often detected in feather clipping studies and attributed to increased energy expenditure [28, 30, 35–41]. However, the possibility of reduced food intake should also be considered (see Discussion).

The present study aims to infer the link between red carotenoid-based ornament expression and flight metabolism by increasing flying effort in wild male common crossbills. In captive males of this species, we have recently described that the treatment with synthetic mito-targeted antioxidant (mitoTEMPO) favored the production of a redder plumage [15]. We have also found that wild male crossbills lose plumage redness with age, which may suggest a link to senescence and, accordingly, oxidative stress [42]. Redder crossbills also have higher probabilities of being recaptured (i.e., a proxy of survival [42, 43]), thus linking plumage color to individual fitness. Importantly, we have recently found that redder males have proportionally longer flying feathers, thus probably correlating flying activity to carotenoid conversion [44].

Here, we hypothesize that red ketocarotenoid-based plumage coloration can signal a certain phenotypic profile that could involve the flying metabolism. In that case, we may predict that increased flying effort could alter (increase or decrease) the conversion rate of substrate yellow to red carotenoids and, consequently, plumage redness. To test the latter, we manipulated the flying capacity by wing feather clipping to test changes in plumage redness after feather growth. We also plucked the rump feathers in all the birds to accelerate plumage regrown, a method previously used in this species (i.e. [15]). We measured the rump color and body mass just before plucking it and when the birds were recaptured several weeks later. We assume that our manipulation increased the flying effort. We should note that crossbills are social birds during the winter [45], clipped individuals being probably forced to increase their energy expenditure to follow the flock. In this regard, the individual body mass change was tested, a body mass loss being predicted in clipped birds but not in controls.

In terms of plumage coloration, two alternative predictions can be formulated. First, a reduced flying capacity reduces plumage redness. This could be expected if wing-feather clipping diminishes the capacity to acquire dietary yellow carotenoids or augments oxidative stress, decreasing carotenoid availability to feathers (e.g. carotenoids being bleached by ROS or consumed as antioxidants) [46, 47]. A hypothetical flight-related oxidative stress could also directly impair ketolase activity, considering that high ROS levels alter the mitochondrial metabolism [20]. Alternatively, the manipulation could increase plumage redness. If joined with a body mass loss, this outcome would agree with a scenario where exercise raises redox rates (often described in mammals; e.g. [48, 49]), favoring enzymatic carotenoid conversion, such as early envisaged by Völker [11].

## Results

### Testing potential initial biases

Among the initially captured birds (*N* = 295), the minimum age in months, initial date of capture and plumage color categories were not significantly biased between treatments (Mann-Whitney’s *U* = 10,232, *p* = 0.379, *U* = 9804, *p* = 0.143 and *χ*^2^ = 3.54, df = 3, *p* = 0.315, respectively; all tests: *n* = 295). Moreover, the wing and tail lengths did not differ (both MW tests *p* > 0.240) and neither the tarsus and head lengths or body mass (*t* = 0.170, df = 286, *p* = 0.865, *n* = 288, *t =* 0.193. df = 286, *p* = 0.847, *n* = 288, and *t* = 0.872, df = 292, *p* = 0.384, *n* = 294, respectively). Size-corrected body mass was also balanced between treatments at the first capture (treatment: *F*_1,281_ = 0.28, *p* = 0.591; tarsus length: *F*_1,281_ = 11.7, *p* < 0.001; wing length: *F*_1,281_ = 13.6, *p* < 0.001; *n* = 285). The muscle and fat scores neither differ (Mann-Whitney’s *U,* both *p* > 0.80, *n* = 295).

The number of crossbills finally recaptured and tested for rump color variability was not biased regarding the initial sample size of each treatment (i.e., 20 control and 14 clipped birds from 151 controls and 144 clipped individuals: *χ*^2^ = 0.89, df = 1, *p* = 0.344). In this subsample (i.e. *n* = 34), no significant difference in rump hue (redness), saturation or brightness was found at the first capture (*U* = 119, *p* = 0.462, *t* = − 0.93, df = 32, *p* = 0.361 and *t* = 0.17, df = 32, *p* = 0.863, respectively). Similarly, no color component of any other body region (i.e. head and back or breast areas) reported any significant bias between treatments (all *t-*tests: *p* > 0.50). Moreover, the minimum age in months, the initial capture date, the number of days elapsed from that date to the recapture, and body morphometries including size-corrected body mass did not significantly differ with treatments in this subset (all tests *p* > 0.20; also Table S1). Only the initial body mass showed a trend to statistical significance (*t* = 1.75, df = 32, *p* = 0.090), with clipped birds tending to be heavier than controls (means± SD: 38.6 ± 1.5 g and 37.4 ± 2.2 g, respectively). However, initial body mass did not correlate with rump redness at the first capture or recapture (Spearman’s *r* = − 0.202, *p* = 0.251 and *r* = 0.08, *p* = 0.654, respectively). Lastly, muscle and fat scores did not differ (Mann-Whitney’s *U,* both *p* > 0.74), and the cloacal protuberance assessed at the first capture showed high incidence among recaptured birds (28/34: 82%) but equal distribution between both treatments (*χ*^2^ = 0.23, df = 1, *p* = 0.628).

### Experimental effects

**Fig. 1:**
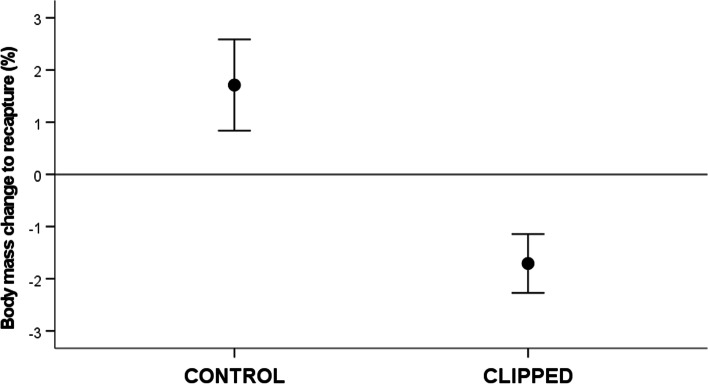
Mean ± S.E.M body mass change (%) from the initial capture to recapture in adult male common crossbills used as controls or manipulated to increase their flying effort by clipping two primary feathers (i.e. third and fifth) of each wing (final body mass: 38.0 ± 0.50 g and 37.8 ± 0.49 g, respectively, for the 20 control and 14 clipped birds recaptured)

The body mass change (%) from the initial to the second capture significantly differed between control and clipped birds (i.e. *t* = 2.97, df = 32, *p* = 0.006; Cohen’s *d* effect size: 1.04; see Fig. 1). Clipped birds lost about 1.7% of their initial body mass (difference to zero: *t* = − 3.03, df = 13, *p* = 0.010), whereas controls showed a statistical trend to increase body mass in a similar proportion (*t* = 1.96, df = 19, *p* = 0.065; Fig. 1). The muscle score at recapture ranged from 1.5 to 3 in the 0–3 scale, and did not differ between treatments: *U* = 114, *p* = 0.348). The subcutaneous fat score did not show variability, being untested (always a 0.5-value, small stripe into the furcular depression).

The treatment did not exert a significant effect on the redness of the head and back or chest (i.e., non-manipulated plumage) at the recapture time controlled for initial variability (see Supplementary Information). However, the rump model reported a significant experimental effect on the redness of the regrown feathers (Table 1).

**Table 1:** ANCOVA testing the effect of the treatment (bold; control vs clipped birds) controlling for several covariates. Body mass change was the difference between final and initial body masses in grams

	Sum of squares	d.f.	Squared mean	***F***	***p***
Intercept	8.636	1	8.636	1.401	0.248
First capture date	8.925	1	8.925	1.447	0.240
Days to recapture	0.014	1	0.014	0.002	0.963
Initial body mass	0.367	1	0.367	0.059	0.809
Body mass change	4.617	1	4.617	0.749	0.395
Tarsus length	17.313	1	17.313	2.808	0.106
Wing length	0.439	1	0.439	0.071	0.792
Initial rump hue	1.088	1	1.088	0.176	0.678
**Treatment**	**27.243**	**1**	**27.243**	**4.418**	**0.046**
Error	154.147	25	6.166		
Total	40,884.583	34			
Total corrected	219.967	33			

**Fig. 2:**
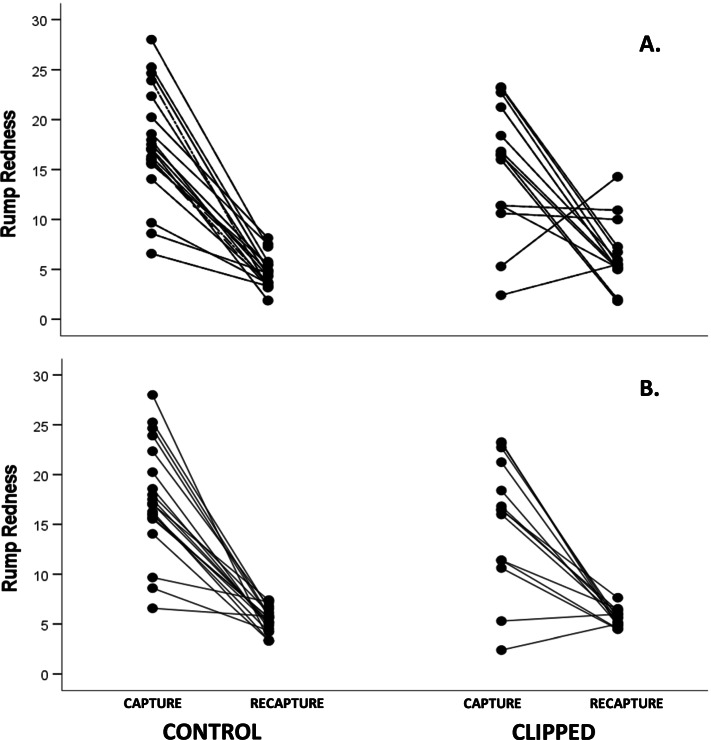
Effect of feather clipping on male common crossbill rump redness at first capture (left) and recapture several weeks later (right). **A** Raw data and (**B**) raw values at capture but predicted values at recapture. Predicted values were obtained from the Table 1 model. Control (non-manipulated wings): *n* = 20. Clipped wings: *n* = 14

Clipped birds showed a redder rump than controls at recapture (Fig. 2A and Fig. S1). However, the rump redness decreased in most birds, the clipped birds showing a weaker decline than predicted (see Fig. 2A vs B). Two birds (both clipped individuals) increased redness from the original rump color (*χ*^2^ = 3.04, df = 1, *p* = 0.081). No covariate reported a significant correlation to final rump redness (Table 1). The treatment factor remained significant when all the covariates are removed (*F*_1,32_ = 4.34, *p* = 0.045). One clipped bird showed a redness value higher than 1.5 x interquartile rank of its treatment and capture event, thus being potentially an outlier (Fig. 2 and also Fig. S1 for interquartile ranks). However, the model reported the same significant treatment effect when it was removed (*F*_1,24_ = 4.50, *p* = 0.045). Moreover, we should note that the model also controls for the initial variability, which was broader (Fig. 2). Furthermore, non-parametric analysis based on ranks also provided a significant difference between the two groups (*U* = 80.5, *p* = 0.037; Supplementary Information).

## Discussion

**Fig. 3:**
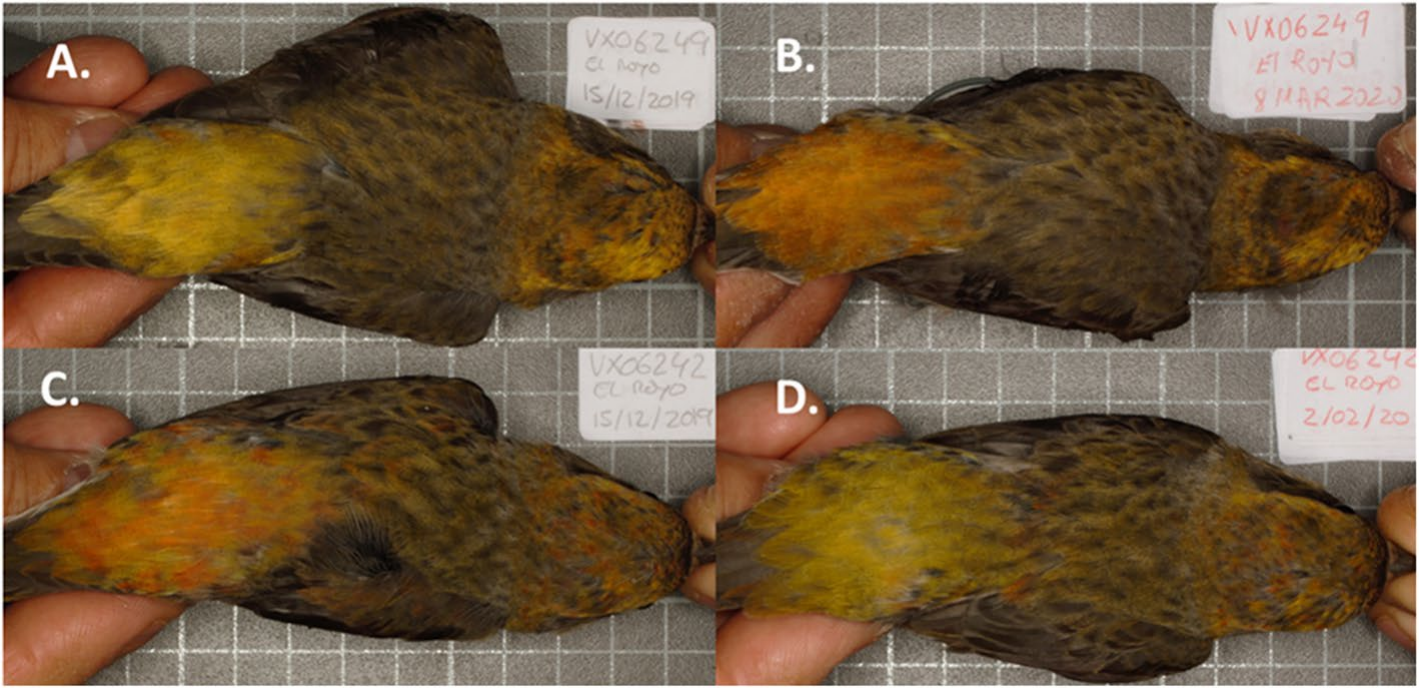
Examples of plumage color variation between capture and recapture dates in a clipped bird (**A**., **B**.) and a control individual (**C**., **D**.) before and after manipulation (from left to right). The new rumps always ranged from yellow to orange colors (see also Fig. 2)

Our results indicate that male crossbills with experimentally impaired flying capacity and, probably, obligated to alter their metabolism (Fig. 1) were able to produce redder feathers than controls (Fig. 2; see examples in Fig. 3). The red coloration of male common crossbills is due to the accumulation of red ketocarotenoids (mainly 3-hydroxyechinenone) in feathers, which requires enzymatic oxidation (ketolation) of yellow dietary carotenoids [14, 15]. Thus, the main result seems to support the hypothesis that flying effort influences carotenoid conversion.

Control male crossbills tended to gain weight during the study (Fig. 1). Increased body mass and fat reserves from winter to spring have been documented in captive and wild common crossbills from North America and attributed to the accumulation of body reserves to prepare for the nomadic movements occurring during the summer [50, 51]. However, this increase has not previously been detected in North Spanish populations [52]. In contrast to controls, clipped crossbills lost body mass. A body mass loss among males that were wing clipped during the breeding period has been reported in many species and mostly interpreted as a consequence of increased flying effort due to reduced lift (e.g. [28, 35, 36, 38, 40, 41]). Reduced body mass was also detected in captive wing-clipped tree sparrows (*Passer montanus* Linnaeus) during their molt, pectoral muscles increasing mass probably due to higher flight activity [37]. Here, fat and muscle scores did not provide any clue, but they reported low variability and were probably insensitive to subtle changes.

Alternatively, the clipped birds could have reduced body mass to compensate for the high flying cost imposed by increased wing loading. Compensatory body mass loss has mostly been described during the nestling-feeding period and interpreted as a way to optimize foraging efficiency (e.g. [53–55]; but see also [37]). Crossbills have the main breeding peak between February and March [52]. Unfortunately, we did not know the reproductive status of each bird. Nonetheless, at first capture, a rough index of active reproduction (i.e. cloacal protuberance occurrence [56]) reported no difference between treatments. Therefore, we cannot infer that clipped individuals were engaged in different body mass regulations, although it cannot be fully discarded. However, we should note that a programmed body mass loss should involve reduced food intake (i.e. programmed anorexia [57]), probably also constraining carotenoid ingestion and limiting pigment conversion. Thus, in our view, the most parsimonious interpretation of reduced body mass in clipped birds is that it was indeed a consequence of higher energy consumption to face flying effort.

Interestingly, the redness value of the new rump was approximately three times lower than that found at the first capture (Fig. 2). The birds were only able to regrown yellowish to orange feathers. We here propose three non-excluding explanations:

First, birds were mostly captured out of the molting season [52, 58], and most of the recaptured birds were probably involved in reproductive investments (see 82% of cloacal protuberance occurrence). Reproduction may entail an important energetic expense and is also linked to higher oxidative stress (e.g. [59, 60]), which could potentially impair the enzymatic redox processes and carotenoid conversion. The high costs linked to reproduction may explain why most avian species avoid molting during the peak of the reproductive effort [61, 62]. Moreover, the natural molt involves a specific endocrine status in birds different from that of reproductive individuals [62], and hormones could influence carotenoid conversion. For instance, high thyroid hormone levels are involved in avian molt activation [63, 64]. In captive male zebra finches (*Taeniopygia guttata* Vieillot), a triiodothyronine (T3) treatment triggered the molt of body feathers [65]. Simultaneously, the bill redness of these birds (a red-ketocarotenoid-based trait [66]) was affected by the interaction of a mitochondria-targeted antioxidant (mitoTEMPO) treatment and T3 dosage [65]. Thus, although the carotenoid-based ornament of zebra finches is not a plumage trait, the result suggests that the mitochondrial function could require a certain endocrine status to favor pigment conversion.

Second, low autumn-winter temperatures could affect redox metabolism. Low winter temperatures increase oxygen consumption and heart rates in common crossbills [67, 68]. A different metabolism could then force a trade-off favoring survival over color expression, perhaps biasing mitochondrial metabolism from cell respiration linked to carotenoid conversion to heat production. Nonetheless, such a mitochondrial uncoupling is still unclear in birds [69, 70].

Third, low availability of dietary substrate carotenoids during winter could have limited carotenoid transformation. In male American redstart (*Setophaga ruticilla* Linnaeus), a hypothetical scarcity of carotenoids in food was also argued when interpreting decreased red chroma detected in a tail feather plucked and regrown during winter [71]. Moreover, in another close-related crossbill (i.e. *Loxia leucoptera* Gmelin), circulating β-cryptoxanthin levels (substrate for red ketocarotenoids in common crossbills; see [14, 15]) decrease out of the molting months, suggesting a decline in the availability of dietary sources of pigments [72].

Whatever the factors constraining carotenoid conversion during the study, the new regrown plumage produced enough color variability to detect a significant experimental effect (Fig. 2). This took place even with the reduced sample size of this study (only 34 birds were recaptured). Contrarily to the prediction that increased flying effort could constrain carotenoid acquisition or impair redox transformations due to oxidative damage, leading to less red plumage, clipped crossbills produced redder (or more orange) feathers than controls. We highlight that, in a similarly short color range (yellow to orange) derived from a plucking experiment under captivity [15], a positive correlation (Spearman’s *r* = 0.53) between crossbill rump redness and feather red ketocarotenoid concentration was detected (also Supplementary Information). This suggests that the effect found here was indeed due to a higher activity of oxidase enzymes in charge of carotenoid biosynthesis (i.e. ketolases; e.g. [73]) among wing-clipped birds. Thus, the result seems to support a link between flight-related metabolism and carotenoid conversion. At first glance, it coincides with Barron et al.’s study [34], where wing-clipped male red-backed fairy-wrens produced larger red plumage areas than controls (the red color is also due to ketocarotenoids in this species [74]). Their analyses focused on recaptured birds (ten controls and ten clipped birds), surprisingly finding that only control birds lost body fat during the study [34]. The authors attributed this to reduced activity but not to higher flight effort. A link to flying metabolism was, thus, not considered. Moreover, the study did not report the number or characteristics of all the wrens initially manipulated or used as controls, potential biases or differential recovery rates being untested.

In the context of animal signaling theory, our result may suggest that common crossbill coloration could inform signal receptors about the intrinsic quality of the bearer in terms of cell metabolism (i.e. [6, 10]). However, it could also reveal a general phenotypic profile, including the flying capacity and other correlated traits linked to fitness. In this line, Chui et al. found that male golden-crowned kinglets (*Regulus satrapa* Lichtenstein) with redder crowns colored by ketocarotenoids left for migration earlier than yellow-crowned individuals [75], probably allowing early reproduction in breeding grounds and better reproductive success (e.g. [76]). In the same line, Mateos-Gonzalez et al. found that redder male house finches (*Haemorhus mexicanus* Müller) were better able to escape from capture into the aviaries than yellowish birds, suggesting that red birds are better at avoiding predation [77]. Note that the house finch plumage is colored by the same red transformed ketocarotenoid (3-hydroxy-echinenone) that male common crossbills deposit on their plumage [9, 14, 15]. Moreover, we have recently found that redder male crossbills have proportionally longer flight feathers than yellow birds [44] and more probabilities of being recaptured in the wild (an index of survival and, hence, of individual quality) [42, 43]. Therefore, if the plumage color of male common crossbill indeed acts as an animal signal, a redder plumage could transmit a better individual quality in adaptation to flight.

## Conclusions

Despite the small sample size due to limitations linked to fieldwork in the studied species, the experimental output seems to suggest that flying effort may indeed influence the production of a redder ketocarotenoid-based coloration. The precedent studies in this and other bird species indicate that increased redness should have implied increased conversion of yellow dietary carotenoids into red ketocarotenoid pigments. Although the experiment involved few individuals, the finding is highly suggestive of a link between flight and red keto-carotenoid conversion leading to color variability. This may contribute to understanding the mechanisms involving the expression of these traits in animals. However, we should note that the experimental approach forced all the clipped birds to afford the challenge during a relatively short time-lapse (a few weeks) during the non-molting season. We tentatively hypothesize that the natural covariation between carotenoid-conversion rates, color production during natural molt and flying capacity could have evolved so that the colored ornament might reveal a general phenotypic profile, including a certain flight-related metabolism. However, it is obvious that more correlational and experimental studies on free-ranging birds must be made to confirm this. Moreover, future works in this or similar animal models should focus on cell respiration metabolism and ketolase activity rates during the production of the colored trait.

## Materials and methods

The manipulation of free-ranging male crossbills required many captures, allowing to recapture of enough experimental and control birds. This was only feasible during autumn and winter when birds group in flocks and crossbill captures rise. In that period, the birds are mostly resident individuals with high recapture probabilities [52, 78]. According to this, the manipulation period was performed from October 19, 2019, to February 14, 2020. This allowed capturing 295 adult male crossbills in the same ringing station (El Royo, Soria, Spain) throughout 17 sampling sessions. This sample size was needed to obtain a minimum recapture sample allowing comparisons. All the birds were captured in that ringing station from two 12 long 16 mm mesh mist-nets placed close to sites traditionally used to provide salt for livestock [58].

The captured birds were ringed, and their sex and age EURING code were notated (based on [79, 80]). The minimum age in months was subsequently established based on the plumage features following [42]. The birds were then weighed (±0.1 g) and several biometric variables were measured with a digital caliper. These included the wing length (± 0.5 mm, method III in [80]), tail length (± 0.5 mm), tarsus length (± 0.01 mm) and head length (± 0.01 mm). Some of these morphometries were not available for some few birds at the first capture (1–6 individuals depending on the trait; detailed in Supplementary Information). The subcutaneous fat extension and pectoral muscle shape were registered in a visual score (fat: 0–8, with 0.25 subclasses [81]; muscle: 0–3 scale with 0.5 subclasses [82]) and always by the same person (DA). The occurrence of cloacal protuberance (a reproduction index) was also registered [56]. Lastly, a four-level score was used to classify each bird’s plumage color, and two digital pictures of each individual were taken (see Color measurements below).

Birds were then assigned to the control or feather-clipped group. In the latter group, primaries 3 and 5 of each wing (counting inward from the distal wing margin) were clipped at the base of the rachis with scissors in feather-clipped males. Control males were similarly handled but without feather clipping. Additionally, the rump feathers of both groups were plucked after taking pictures (see below) to stimulate plumage regrown. The group assignation was a priori made randomly. However, we also attempted to balance the treatments regarding the cited plumage color categories and the bird’s age (please, see Supplementary Information). Thus, finally, 151 controls and 144 clipped-feather birds were assigned. Only three controls and one clipped bird were ending their wing molt at the time of manipulation (treatment x molt occurrence: *χ*^2^ = 0.92, df = 1, *p* = 0.337, *n* = 295). Among these four birds, only one control bird was recaptured. The results were similar when excluding these four individuals from the analyses.

Recaptures took place from January 2 to July 24, 2020. This means some birds could be recaptured during the period dedicated to treatment assignment and manipulation of other birds. Only those recaptures that took place more than three weeks after the first capture were included in the statistical analyses. This was the minimum period observed to allow birds to regrow their rump feathers fully. Five recaptures produced within less than three weeks were accordingly discarded as the rump was not regrown (lapse mean ± S.D.: 81 ± 9.3 d, range 21–208 d). The first birds showing a natural molt of the rump were found after July 24. Thus, only the color of the feathers induced by the experimental plucking was analyzed—a full inspection of body plumage from pictures and wing feather molting patterns allowed to verify this. Thirty-four recaptured birds met these criteria (20 control and 14 feather-clipped birds).

### Color measurements

Digital photographs of the breast and the back of red crossbills were always taken by putting the birds at the same position and fixed distance from the objective (Canon Macro Lens EF 50 mm; see also [15] and Fig. 3). A Kaiser Repro Base (Kaiser Fototechnik, Buchen) was used, including a gridded board and a column to place the camera at the same height (distance from the board to the lens: 38 cm). The base was covered with opaque grey cellular polycarbonate sheets placed vertically to cover the four sides of the board. It allowed us to enter the camera objective and ring flash from the top (Canon Macro Ring Lite MR-14EX). The sheets were perforated to allow entering the hands of a person that would hold the bird’s body resting on the board surface. All the open surfaces were covered with PVC blackout fabric to reduce the light on the board surface. The focus and diaphragm of the camera and the ring flash were all manually fixed to avoid the interference of automatic functions.

Two digital pictures of each bird were taken (i.e. one including the breast and abdomen, and another the backside). Thus, the bird was placed in face-up or prone positions by pulling the bill and legs. A standard grey card (Kodak, NY, USA) was used as a reference, placed next to the bird’s flank on the board surface, and always in the same position. Digital photographs were standardized and analyzed using ‘SpotEgg’ software [83], which allows the user to manually draw any region and provide information about its coloration, size or shape. Areas with disorganized feathers were avoided. For each individual, the average of red, green, and blue components of the colored area of the chest (1), the head and back (2), and rump (3) were separately calculated. The rump area in the picture of the first capture was similar to that measured on the new rump and highly correlated (Pearson’s *r* = 0.83, *p* < 0.001). We then determined each area’s hue (°), chroma and brightness through the Foley and van Dam algorithms [84]. In a previous study on the same species, the repeatability [85] of these three variables taken twice was very high (all *r* > 0.90, *n* = 30) [15]. Finally, since a low hue indicates a redder color, we reversed their values (i.e. multiplying by − 1) to obtain a more intuitive variable, i.e. “redness.” We rescaled the variable by adding the minimum negative value to obtain positive data. Thus, high plumage redness values indicate redder traits [15, 65]. The rump hue measures taken from another sample of crossbills reported a high correlation to red ketocarotenoid concentrations in rump feathers (Supplementary Information).

In addition, the birds were also classified in one of the four-color categories described by del Val et al. [86], i.e., yellow, patchy, orange or red. These categories consider the whole body area [42]. This body color score allowed to balance the treatment assignation in terms of individual coloration. The score correlates with objective colorimeter measures [86] and is repeatable when assessed twice by the same person (DA) naïve to bird identity (measures separated by one week; see [15]). Moreover, the score also positively correlated with the concentration of red ketocarotenoids in feathers but not with yellow carotenoid levels (Supplementary Information).

### Statistical analyses

The analyses were performed with SPSS version 27. In the initial capture, the sampling date, minimum age (i.e. [42]), and wing and tail did not follow a Gaussian distribution (Shapiro-Wilk tests), and arithmetic procedures could not normalize them. Accordingly, treatment comparisons were made by non-parametric Mann-Whitney’s U tests in these variables. The body mass, head and tarsus length were normally distributed, and accordingly, comparisons were tested by Student’s t-tests. An ANCOVA model testing initial body mass as the dependent variable, the treatment as a fixed factor, and morphometries as covariates was also performed to test potential biases in size-corrected body mass at the first capture (only covariate terms at *p* < 0.10 were retained in the model after a backward stepwise procedure).

In the dataset only including the recaptured birds (i.e. *n* = 34), the initial date of capture, number of days elapsed to the recapture and tail length were not normally distributed and compared by Mann-Whitney’s U tests. The initial body mass, body mass change to recapture (%) and other morphometries followed a Gaussian distribution and were analyzed by Student’s *t*-tests. Size-corrected body mass was tested using an ANCOVA, testing body mass as the dependent variable, the treatment as a fixed factor and significant body morphometries as covariates. Also, in this subset, the hue, chroma or brightness at the first or second capture dates, and in any body part, were normally distributed. However, the hue of the rump did not follow a normal distribution, and arithmetic transformations could not normalize it. Moreover, the residuals of parametric models, including different covariates (ANCOVA), were always non-normal (all Shapiro-Wilk tests: *p* < 0.01). However, some authors consider that lack of normality could not be enough to reject the use of parametric analyses [87]. Therefore, we performed parametric models including the treatment as a fixed factor and different covariates to control for subtle confounding influences (see Table 1). In any event, the hue of the rump was also tested by non-parametric tests reporting the same significant treatment effect (see the Supplementary Information). Parametric ANCOVAs were also used to test the hue of the chest and head and back surfaces. This would allow to infer if abrasion or color bleaching due to sunlight (e.g. [61]) could have been biased among experimental groups, influencing the results of the rump redness (see Supplementary Information). Finally, bivariate Spearman’s correlation coefficients were used when at least one of the variables was not normal. Two-tailed tests were always used as we predicted alternative outputs (Introduction).

## Supplementary Information

**Additional file 1.**

**Additional file 2.**

## Data Availability

All data generated or analyzed during this study are included in this published article [and its supplementary information files].

## Abbreviation

IMM: Inner mitochondrial membrane
