# Supplementary material for: Wild common crossbills produce redder body feathers when their wings are clipped
Source: BMC Zool. 2022 Aug 23;7:47. doi: 10.1186/s40850-022-00150-9 (PMC10127331; doi:10.1186/s40850-022-00150-9)
Supplement: Supplementary file 2 — Additional file 2. [file 40850_2022_150_MOESM2_ESM.docx]

**SUPPLEMENTARY INFORMATION**

**Legends of Supplementary Information**

**Supplementary Information doc.** This is the present text that reports methodological details and complementary analyses and figures.

**DATASETS.** This is an excel file (book) including all the data used in the statistical analyses. The first sheet shows the data from the first capture of birds and the second one reports the data for recaptured individuals only.

**METHODOLOGICAL DETAILS**

**Treatment assignation**

The group assignation was a priori made randomly. However, we also attempted to balance the treatments regarding the cited plumage color categories and the bird's age. Regarding the color, this was made by visually assigning one of four different plumage color categories (see below), alternating the treatment (color vs clipped) within each color category at the time of capture. During the captures, we also regularly checked if some age category (EURING codes) was not well represented in any of the two treatments. In that case, we tried to compensate for this by assigning birds to the scarcest group at that age.

**Additional comparisons of morphometric among recaptured birds**

We compared the morphometric variables and body mass of control and treated crossbills among the recaptured individuals only, reporting no significant difference.

Table S1: Parametric Student’s *t*-tests of the difference in morphometry and initial body mass between recaptured control and clipped crossbills. The size-corrected initial body mass was analyzed as the standardized residual of body mass on wing and head length (only body size covariates at *p* < 0.10). No test reached significance (see also main text). Tail length (not normally distributed) was also tested by non-parametric Mann-Whitney test: *U* = 121, *p* = 0.496).

|  | ***t*** | ***df*** | ***p*** |
| --- | --- | --- | --- |
| Wing length | 0.708 | 32 | 0.484 |
| Tail length | 0.631 | 32 | 0.533 |
| Tarsus length | 0.501 | 32 | 0.62 |
| Head length | 1.035 | 32 | 0.308 |
| Initial body mass | 1.75 | 32 | 0.09 |
| Size-corrected initial body mass | 1.67 | 32 | 0.206 |

**Morphometric data absent in some few birds**

Among the 295 adult males captured and involved in the experiment (i.e. 151 controls and 144 clipped birds), some morphometric data were not available at the first capture event due to oversight or being illegible. This involved the body mass of one control bird, the wing length of two controls, the tail length of another control, and the head and simultaneously the tarsus length of four control and three clipped birds. None of the cited birds was included in one of the other lists here described. Data absence did not take place among recaptured birds.

**Plumage hue as a proxy of red carotenoid concentration in feathers**

The hue was chosen as a good proxy of color variability and ketocarotenoid concentration in feathers. In a recent experimental study, we analyzed carotenoid pigments' concentration in male common crossbills' rump feathers [1]. In that study, the rump feathers were plucked in the same way described in the current work. We tested correlations between rump color components (hue, saturation, and brightness) and carotenoid concentrations in the same feathers. We found that the hue was negatively correlated (i.e. positively related to redness) to the sum of all red ketocarotenoids in feathers (i.e. 3-hydroxy-echinenone, astaxanthin, canthaxanthin and echinenone; Spearman’s correlation coefficient: *r* = -0.53, *p* <0.001, *n* = 41). In contrast, the saturation and brightness did not correlate with the cited variable (*r* = -0.19, *p* = 0.907 and *r* = -0.18, *p* = 0.272, respectively; also *n* = 41). No significant correlation was found in the case of the total concentration of yellow pigments (lutein plus β-cryptoxanthin) in the same feathers (all *p* > 0.1 for the three color components). The hue was also correlated to 3-hydroxy-echinenone (the main red carotenoid in crossbills’ feathers; Cantarero et al., 2020) when tested alone (*r* = -0.52, *p* < 0.001), the saturation and brightness again reporting non-significant tests (*r* = 0.031, *p* = 0.846 and *r* = -0.16, *p* = 319). Similar findings were found when using parametric Pearson's coefficients. However, carotenoid levels did not fit well with a normal distribution [1]; hence, Pearson's r values are not reported here. We can conclude that hue is a better indicator of feather carotenoid variability than saturation or brightness.

**The body color score also correlated to rump carotenoid levels.**

The body color score and carotenoid concentrations in rump feathers were correlated in an independent dataset from an unpublished study (*N* = 200; Alejandro Cantarero, Blanca Fernandez-Eslava, Daniel Alonso, Pablo Camarero, Rafael Mateo and Carlos Alonso Alvarez, unpublished material). The rump of patchy birds, as expected, included yellow and orange feathers. Carotenoid concentrations were assessed by high precision liquid chromatography (HPLC) following [1]. The four-level score was determined as described in the main text (**Color measurements** in Material and Methods). The concentration of 3-hydroxy-echinenone or the sum of all red ketocarotenoids in feathers were positively correlated to the body color score (Spearman’s *r* = 0.74 and 0.75, respectively, both *P* < 0.001). Instead, the sum of the two yellow carotenoids in feathers (lutein plus β-cryptoxanthin) did not correlate to the score (*r* = -0.017, *P* = 0.82).

**ALTERNATIVE STATISTICS AND COMPLEMENTARY ANALYSES**

**Alternative non-parametric test of color differences**

Alternative non-parametric tests based on ranks less sensitive to outliers showed that the redness of the rump strongly declined from the first to the second capture (see Fig. S1 and Fig. 2 of the main text). Thus, Wilcoxon matched-pairs tests showed: *Z* = -3.92, *p* < 0.001 and *Z*= -2.79, *p* < 0.001, for control and clipped birds, respectively). The redness of the regrown rump was uncorrelated to the original value (Spearman’s *r* = 0.126, *p* = 0.477). This could be due to reduced redness variability (SD dropped from 5.95 to 2.98) and the experimental effect (Fig. S1). Thus, a positive correlation seems to arise among controls but not in clipped birds (Spearman’s *r* = 0.395, *p* = 0.085 and *r* = -0.125, *p* = 0.669, respectively). Despite the evident color decline and lack of within-individual correlation, the redness of the new regrown feathers significantly differed between treatments (*U* = 80.5, *p* = 0.037), with clipped birds developing a redder rump than controls (Fig. S1 and Fig. 2 in the main text). Therefore, non-parametric tests agree with those parametric analyses described in the main text.

**
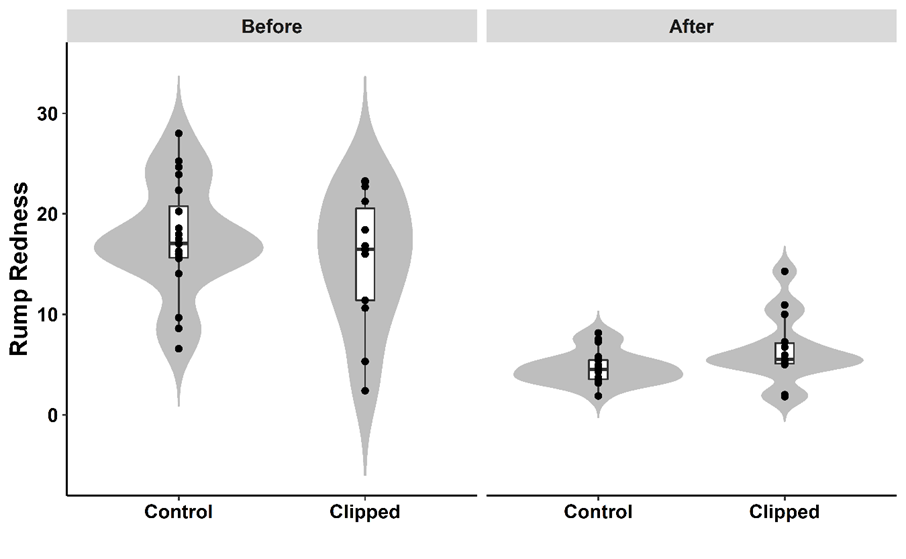
**

**Fig. S1.** Effect of feather clipping on male common crossbill rump redness. Raw values (dots) of rump redness before (left) and after (right) wing feather clipping (20 control and 14 clipped birds recaptured). The violin plot represents the density function. Medians, quartiles, and ranges are shown (see non-parametric statistics above).

**The change in color of other non-manipulated body regions**

The final hue obtained from the head and back surfaces (as a whole, excluding the rump) or the chest surface were used as non-manipulated (control) body areas to test if feather abrasion or color bleaching with time among controls could potentially have biased the rump result. The experimental treatment was not significant in both the head and back redness (treatment: *F* _1,31_ = 0.52, *p* = 0.821; initial redness covariate: *F* _1,31_ = 193.7, *p* < 0.001) or chest (treatment: *F* _1,31_ = 2.30, *p* = 0.139; initial redness covariate: *F* _1,31_ = 319.6, *p* < 0.001). The saturated model led to similar conclusions. Note that the initial hue variability strongly correlated to the final hue as no manipulation (feather plucking) was made (the feathers were the same). In addition, when the standardized residuals of the final hue on initial hue in both surfaces or the difference between final and initial hue values (final minus initial) were included as alternative covariates in the Table 1 model none of them reported a significant correlation to rump redness (all *p-*values > 0.40).

**References**

Cantarero A, Mateo R, Camarero PR, Alonso D, Fernandez-Eslava B, Alonso-Alvarez C. Testing the shared-pathway hypothesis in the carotenoid-based coloration of red crossbills. Evolution. 2020;74:2348-64.
